# Supplementary material for: Characterization of Dissolved Organic Matter in Solar Ponds by Elemental Analysis, Infrared Spectroscopy, Nuclear Magnetic Resonance and Pyrolysis–GC–MS
Source: Int J Environ Res Public Health. 2022 Jul 25;19(15):9067. doi: 10.3390/ijerph19159067 (PMC9331927; doi:10.3390/ijerph19159067)
Supplement: Supplementary file 1 [file ijerph-19-09067-s001.zip › ijerph-1806067-supplementary.pdf]

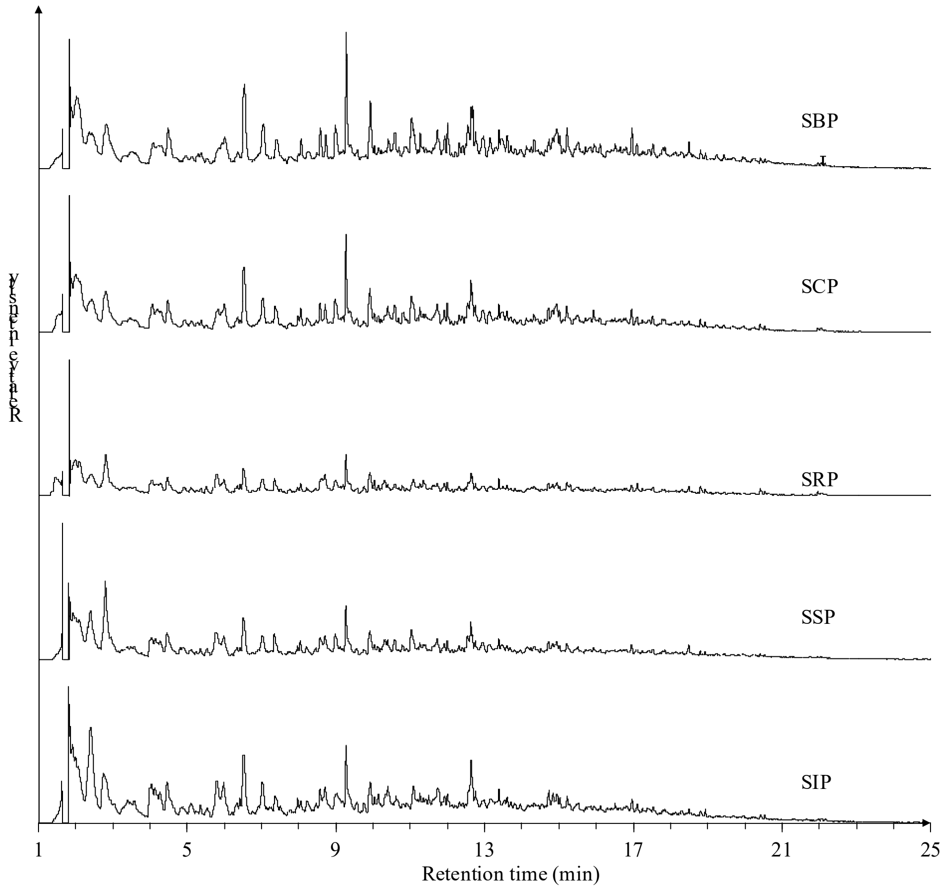

Figure S1. Total ion chromatograms of pyrolysis products of DOM samples

Table S1. Pyrolysis product of SPE-DOM isolated from SS samples with corresponding retention times (RT), ion fragments used for quantification (m/z), component group and relative proportions (% of total quantified peak area, sum 100%). Abbreviations: ALICYCL= alicyclic compounds, CARB = carbohydrate, MAH= monocyclic aromatic hydrocarbon, MCC= methylene chain compound, NCOMP= nitrogen-containing compound, PAH= polycyclic aromatic hydrocarbon, PHEN = phenols, SCOMP= sulphur-containing compound, PENT= Pentanedioic acid, LIG = products of lignin and lignin-like phenolics; T=tentative identification.

RT	Compound	m/z	Class	SIP	SSP	SRP	SCP	SBP
1.86	S2/SO2	64.00	SCOMP	2.05	0.54	1.07	0.61	0.52
1.85	bromomethane	94+96	XCOMP	1.09	0.55	0.27	0.45	0.27
2.08	cyclopentene	67+68	ALICYCL	1.73	2.46	1.83	1.58	1.43
2.00	iodomethane	142+127	XCOMP	0.57	0.12	0.20	0.81	0.85
2.21	trimethylsilanol	75(76,90,45/47)	OTHER	0.09	0.40	0.13	0.16	0.12
2.35	2-propenal	56+55	CARB	1.86	2.38	1.63	1.66	1.34
2.45	acetic acid	45+60	CARB	10.33	4.81	8.66	5.14	4.95
2.43	2-methylfuran	82+53	CARB	2.34	1.97	2.31	1.36	1.17
2.77	cyclohexadiene	79+80(77)	ALICYCL	4.77	4.03	5.46	5.35	4.37
3.00	hydroxypropanone	74.00	CARB	0.06	0.09	0.11	0.08	0.07
2.93	benzene	78.00	MAH	0.64	0.88	0.67	0.71	0.62
3.33	3-hydroxypropanal	74.00	CARB	0.44	0.31	0.50	0.37	0.31

3.23	alkene Cx	55.00	MCC	0.50	0.74	0.59	0.53	0.44
3.38	alkane Cx	57.00	MCC	0.65	0.67	0.70	0.68	0.63
3.49	C1-cyclohexene (T)	81+96	ALICYCL	1.24	1.16	1.42	1.28	1.32
3.49	1,4-dioxane (T)	88 (55)	OTHER	0.02	0.04	0.02	0.01	0.01
3.61	unidentified compound	69+100	OTHER	0.13	0.18	0.14	0.16	0.20
4.02	C1-cyclohexadiene	79+94 (77,91)	ALICYCL	2.98	2.57	2.98	2.71	1.88
4.25	C1-cyclohexadiene	79+94 (77,91)	ALICYCL	2.30	2.05	2.65	2.64	2.07
4.35	C1-cyclohexadiene	79+94 (77,91)	ALICYCL	1.50	1.38	1.73	1.61	1.41
4.65	C1-cyclohexadiene	79+94 (77,91)	ALICYCL	0.67	0.60	0.53	0.44	0.39
4.29	N-methylpyrrole	80+81	NCOMP	0.47	0.59	0.60	0.74	0.82
4.29	pyrrole	67.00	NCOMP	0.66	0.59	0.34	0.38	0.44
4.42	2,3-dihydrothiophene (T)/pentanal	85+86	OTHER	0.03	0.00	0.03	0.02	0.02
4.45	toluene	91+92	MAH	4.50	4.42	5.15	4.88	4.95
5.36	tetramethylcyclopentene compound (T)	109 (67,124)	OTHER	0.21	0.16	0.22	0.27	0.32
4.63	(3H)-furan-2-one	84+55	CARB	0.11	0.04	0.07	0.04	0.05
4.91	unidentified carbohydrate	55+84	CARB	0.25	0.30	0.41	0.28	0.28

4.98	acetic anhydride/2-hydroxybutanaldehyde	55+56 (84,102)	CARB	0.62	1.03	0.85	0.88	0.85
5.13	(2H)-furan-3-one	84+54	CARB	0.32	0.23	0.21	0.09	0.08
5.08	unidentified carbohydrate	95+110	CARB	0.49	0.60	0.62	0.74	0.96
5.62	unidentified carbohydrate	95+67(110)	CARB	0.13	0.47	0.28	0.24	0.27
5.44	unidentified carbohydrate	110+109	CARB	0.28	0.22	0.30	0.36	0.41
5.85	2-propylfuran(T)	110(53)	CARB	0.23	0.21	0.19	0.19	0.14
8.74	5-methyl-2-furaldehyde	110+109(53)	CARB	0.62	0.62	0.51	0.36	0.28
5.80	unidentified carbohydrate	82.00	CARB	0.66	1.52	1.12	0.82	0.40
5.34	3-furaldehyde	95+96	CARB	0.29	0.36	0.33	0.38	0.49
5.80	2-furaldehyde	95+96	CARB	1.30	1.30	1.48	0.97	0.80
5.18	C2-cyclohexadiene	91+93(108)	ALICYCL	0.31	0.28	0.29	0.30	0.23
5.43	C2-cyclohexadiene	91+93(108)	ALICYCL	0.19	0.19	0.16	0.14	0.15
5.55	C2-cyclohexadiene	91+93(108)	ALICYCL	0.13	0.13	0.09	0.08	0.08
5.68	C2-cyclohexadiene	91+93(108)	ALICYCL	0.12	0.15	0.13	0.10	0.10
5.98	C2-cyclohexadiene	91+93(108)	ALICYCL	0.66	0.66	0.64	0.65	0.64
6.43	C2-cyclohexadiene	91+93(108)	ALICYCL	2.92	2.97	3.10	3.88	3.91

6.53	C2-cyclohexadiene	91+93(108)	ALICYCL	2.97	2.92	3.15	3.93	4.09
6.92	C2-cyclohexadiene	91+93(108)	ALICYCL	1.13	1.28	1.29	1.64	1.75
7.24	C2-cyclohexadiene	91+93(108)	ALICYCL	0.31	0.31	0.31	0.27	0.27
7.40	C2-cyclohexadiene	91+93(108)	ALICYCL	0.24	0.25	0.24	0.20	0.23
5.82	2-cyclopenten-1-one	82+53	CARB	0.71	1.50	1.15	0.83	0.44
5.97	C1-methylpyrrole	80+81	NCOMP	0.07	0.07	0.07	0.07	0.07
6.17	C1-methylpyrrole	80+81	NCOMP	0.05	0.00	0.02	0.00	0.02
6.02	unidentified compound	70(55,126)	OTHER	0.07	0.07	0.08	0.09	0.12
6.32	unidentified carbohydrate	54+98	CARB	0.07	0.08	0.04	0.06	0.06
6.23	2-(hydroxymethyl)furan (T)	55+98	CARB	0.17	0.22	0.25	0.31	0.35
6.59	1-pentene-3,4-dione (T)	55+98	CARB	0.29	0.06	0.14	0.10	0.10
6.33	C2-benzene	91+106	MAH	2.14	2.47	2.27	2.69	2.14
6.52	C2-benzene	91+106	MAH	2.43	2.16	2.52	3.22	3.72
6.87	C4-furan (C8H12O)	95 (124?)	CARB	0.14	0.21	0.19	0.19	0.26
6.94	unidentified compound	109+124	OTHER	0.16	0.17	0.20	0.23	0.29
7.31	unidentified compound	109+124	OTHER	0.15	0.16	0.17	0.18	0.20

7.39	C5H12O	68+96 (67)	CARB	0.23	0.29	0.31	0.30	0.25
6.98	styrene	104+78	MAH	0.29	0.19	0.23	0.14	0.22
7.02	C2-benzene	91+106	MAH	1.23	1.23	1.28	1.52	1.71
7.24	C3-cyclohexadiene	107+122	ALICYCL	0.21	0.24	0.20	0.17	0.18
7.53	C3-cyclohexadiene	107+122	ALICYCL	0.16	0.16	0.17	0.13	0.14
7.86	C3-cyclohexadiene	107+122	ALICYCL	0.27	0.34	0.30	0.28	0.27
8.07	C3-cyclohexadiene	107+122	ALICYCL	0.21	0.21	0.20	0.20	0.19
8.31	C3-cyclohexadiene	107+122	ALICYCL	0.22	0.25	0.22	0.22	0.24
9.74	C3-cyclohexadiene	107+122	ALICYCL	0.22	0.27	0.21	0.19	0.17
9.88	C3-cyclohexadiene	107+122	ALICYCL	0.09	0.12	0.10	0.09	0.12
7.34	C1-cyclopenten-1-one	67+96	CARB	0.85	0.73	0.90	1.10	1.38
7.53	2-acetylfuran	95+110	CARB	0.31	0.54	0.34	0.26	0.34
7.60	C12-isoprenoid alkanone (5,8-Decadien-2-one, 5,9-dimethyl-)	107+122	MCC	0.13	0.10	0.13	0.10	0.10
7.68	(5H)-furan-2-one	55+84	CARB	0.21	0.33	0.34	0.20	0.18
7.87	unidentified compound	83 (56,55,69,126)	OTHER	0.07	0.06	0.08	0.09	0.11
7.98	unidentified compound	68.00	OTHER	0.27	0.38	0.41	0.35	0.29

8.19	unidentified carbohydrate	55+98	CARB	0.13	0.08	0.11	0.05	0.02
8.06	3-methyl-2,5-furandione	67+110(95)	CARB	0.49	0.69	0.75	0.81	0.85
8.28	unidentified compound	68 (112)	OTHER	1.05	0.25	0.42	0.48	0.51
8.73	2-methyl-2-cyclopenten-1-one	96 (53,67,81)	CARB	0.32	1.09	0.67	0.28	0.14
8.58	C3-benzene	105+120	MAH	0.66	0.70	0.68	0.87	0.97
8.72	C3-benzene	105+120	MAH	0.18	0.23	0.16	0.19	0.20
9.28	C3-benzene	105+120	MAH	0.47	0.32	0.36	0.30	0.65
9.93	C3-benzene	105+120	MAH	0.47	0.36	0.42	0.37	0.76
10.14	C3-benzene	105+120	MAH	0.20	0.18	0.12	0.10	0.11
10.90	C3-benzene	105+120	MAH	0.13	0.17	0.13	0.17	0.21
9.03	unidentified carbohydrate	110+68 (54)	CARB	0.47	0.30	0.29	0.23	0.22
9.04	4-oxo-pentanoic acid ME (T)	99 (115, 130)	PENT	0.02	0.11	0.05	0.02	0.00
9.00	phenol	94.00	PHEN	1.00	1.01	1.03	1.00	1.01
9.20	benzonitrile	103+76	NCOMP	0.22	0.25	0.25	0.32	0.34
9.05	butanedioic acid diME or 2-methoxy-4-methyl-2-pentenoic acid methyl ester (T)	115 (55)	PENT	0.03	0.00	0.03	0.03	0.00
10.03	butanedioic acid diME or 2-methoxy-4-methyl-2-pentenoic acid methyl	115 (55)	PENT	0.06	0.06	0.06	0.07	0.09

	ester (T)							
11.18	butanedioic acid diME or 2-methoxy-4-methyl-2-pentenoic acid methyl ester (T)	115 (55)	PENT	0.13	0.17	0.14	0.20	0.22
10.21	alpha-methylstyrene	117+118(115)	CONTA	0.12	0.10	0.14	0.15	0.19
10.37	unidentified compound	83 (55?)	OTHER	0.13	0.11	0.12	0.11	0.12
9.71	unidentified compound (C1-cyclohexanone)(T)	(68)+112	OTHER	0.03	0.00	0.01	0.00	0.03
9.83	unidentified compound	(69)+113 (59,84)	OTHER	0.00	0.00	0.01	0.00	0.00
11.07	C3-benzene	91+120	MAH	0.27	0.34	0.29	0.36	0.41
9.54	C10-isoprenoid alkadiene	123 (138)	OTHER	0.09	0.05	0.10	0.08	0.14
9.87	unidentified compound	56 (79,...136)	OTHER	0.07	0.04	0.08	0.06	0.08
9.98	unidentified carbohydrate	95+110(67)	CARB	0.26	0.47	0.38	0.33	0.27
9.97	unidentified compound	70 (110)	OTHER	0.07	0.03	0.06	0.04	0.06
10.19	limonene	67+68 (93,136)	ALICYCL	0.26	0.60	0.38	0.37	0.40
10.40	2,3-dimethylcyclopent-2-en-1-one	67+110	CARB	0.92	0.82	1.04	1.27	0.92
10.40	indene	115+116	PAH	0.05	0.02	0.04	0.03	0.04
10.75	unidentified carbohydrate	82+110 (54)	CARB	0.20	0.28	0.24	0.24	0.20
10.68	C1-phenol	107+108	PHEN	0.32	0.24	0.19	0.19	0.22

11.18	C1-phenol	107+108	PHEN	0.97	1.08	1.13	1.23	1.38
10.89	trimethylcyclopentenone (T)	109+124 (81)	CARB	0.38	0.33	0.44	0.55	0.66
11.47	trimethylcyclopentenone (T)	81+109 (124)	CARB	0.36	0.41	0.41	0.52	0.41
11.09	C4-benzene	119+134	MAH	0.45	0.57	0.50	0.72	0.88
11.28	C4-benzene	119+134	MAH	0.25	0.23	0.23	0.24	0.44
11.93	C4-benzene	119+134	MAH	0.43	0.61	0.51	0.79	1.04
12.03	C4-benzene	119+134	MAH	0.17	0.12	0.13	0.10	0.23
12.34	C4-benzene	91+134	MAH	0.26	0.32	0.25	0.29	0.33
12.69	C4-benzene	119+134	MAH	0.52	0.83	0.66	0.98	1.37
11.34	C4:1-benzene	117+132	MAH	0.33	0.33	0.33	0.38	0.42
11.55	C4:1-benzene	117+132	MAH	0.17	0.22	0.14	0.19	0.20
10.86	C9-isoprenoid alkadiene (2,4-Heptadiene, 2,4-dimethyl-)	109+124 (81)	MCC	0.39	0.48	0.52	0.63	0.65
11.43	guaiacol (T)	109+124 (81)	LIG	0.30	0.40	0.36	0.43	0.32
11.77	C1-benzofuran	131+132	OTHER	0.26	0.23	0.24	0.27	0.28
11.71	unidentified compound	79+122	OTHER	0.43	0.44	0.43	0.50	0.50
11.83	unidentified compound	67+112 (140?)	OTHER	0.51	0.24	0.31	0.37	0.48

12.21	Isoprenoid ketone (e.g.2,4-heptadienal, 2,4-dimethyl-	109+138	MCC	0.10	0.14	0.13	0.11	0.13
12.28	Pentanedioic acid, dimethyl ester	59+100+101+129	PENT	0.08	0.44	0.19	0.08	0.01
12.63	C1-indene/dihydronaphthalene	130+115	PAH	0.41	0.38	0.45	0.60	0.60
12.76	C1-indene/dihydronaphthalene	130+115	PAH	0.08	0.00	0.00	0.00	0.00
12.36	unidentified compound	124 (67,81,109)	OTHER	0.13	0.19	0.17	0.18	0.14
12.63	C2-phenol	107+122	PHEN	0.38	0.23	0.17	0.19	0.23
12.98	C2-phenol	107+122	PHEN	0.37	0.40	0.37	0.45	0.51
13.15	C2-phenol	107+122	PHEN	0.22	0.17	0.13	0.13	0.15
13.62	C2-phenol	107+122	PHEN	0.25	0.26	0.18	0.23	0.26
13.37	naphthalene	128.00	PAH	0.11	0.08	0.09	0.12	0.15
13.40	C5:1-benzene	131+146	MAH	0.27	0.35	0.29	0.47	0.57
13.60	C5:1-benzene	131+146	MAH	0.16	0.15	0.14	0.17	0.16
13.73	unidentified compound	57+69+100	OTHER	0.10	0.14	0.10	0.09	0.09
13.58	unidentified compound	95.00	OTHER	0.14	0.19	0.15	0.18	0.19
14.58	unidentified compound	95.00	OTHER	0.12	0.14	0.12	0.14	0.15
13.78	C3-phenol	121+136	PHEN	0.13	0.10	0.08	0.09	0.11

13.80	C1-benzofuran	145+146	OTHER	0.11	0.11	0.11	0.17	0.18
13.94	C1-benzofuran	145+146	OTHER	0.21	0.19	0.19	0.29	0.30
14.18	benzothiazole	135 (108)	SCOMP	0.14	0.21	0.11	0.08	0.14
14.33	C5-benzene	133+148	MAH	0.09	0.09	0.08	0.11	0.12
15.25	C5-benzene	133+148	MAH	0.08	0.08	0.07	0.09	0.12
14.56	C9H13N (C3-aniline)(T)	135+106	NCOMP	0.16	0.00	0.12	0.08	0.10
16.10	C10H15N (C4-aniline)(T)	134 (106,149)	NCOMP	0.03	0.00	0.03	0.02	0.04
14.71	C2-indene	129+144	PAH	0.85	0.93	0.88	1.26	1.41
14.83	C2-indene	129+144	PAH	0.64	0.68	0.68	0.96	1.17
14.93	C2-indene	129+144	PAH	0.34	0.34	0.35	0.46	0.58
15.02	C2-indene	129+144	PAH	0.12	0.15	0.12	0.17	0.19
15.37	unidentified compound	131+132	OTHER	0.13	0.16	0.15	0.17	0.24
15.56	C3-benzofuran	145+160	OTHER	0.13	0.19	0.16	0.25	0.33
15.80	C3-benzofuran	145+160	PAH	0.11	0.08	0.09	0.00	0.16
16.65	unidentified compound	135+150	OTHER	0.12	0.11	0.12	0.11	0.16
16.95	C3-indene/C2-dihydronaphthalene	143+158 (128)	PAH	0.23	0.25	0.22	0.28	0.39

17.07	C3-indene/C2-dihydronaphthalene	143+158 (128)	PAH	0.13	0.13	0.11	0.13	0.18
17.50	C3-indene/C2-dihydronaphthalene	143+158 (128)	PAH	0.18	0.22	0.17	0.21	0.27
17.52	C2-naphthalene	141+156	PAH	0.15	0.27	0.20	0.33	0.36
17.83	C2-naphthalene	141+156	PAH	0.11	0.10	0.08	0.10	0.15
18.03	C2-naphthalene	141+156	PAH	0.07	0.09	0.07	0.12	0.12
18.21	C2-naphthalene	141+156	PAH	0.10	0.12	0.11	0.16	0.19
15.81	phthalic anhydride	104+76(148)	OTHER	0.04	0.06	0.05	0.11	0.12
5.48	unidentified alkane	57.00	MCC	0.04	0.07	0.07	0.03	0.05
10.15	unidentified compound	56.00	OTHER	0.19	0.14	0.13	0.14	0.13
11.28	unidentified compound	56.00	OTHER	0.12	0.19	0.10	0.11	0.09
5.98	unidentified aliphatic	55+70	MCC	0.08	0.11	0.11	0.11	0.13
11.82	alkanal compound	55+70	MCC	0.10	0.00	0.05	0.03	0.06
12.89	alkanal compound	55+70	MCC	0.06	0.02	0.05	0.03	0.05
11.19	unidentified aliphatic	69+55	MCC	0.21	0.26	0.21	0.16	0.16
15.69	unidentified aliphatic	69+55	MCC	0.13	0.20	0.11	0.11	0.11
16.01	unidentified aliphatic	69+55	MCC	0.12	0.18	0.11	0.10	0.10

16.99	unidentified aliphatic	55+69	MCC	0.21	0.45	0.21	0.27	0.22
19.49	unidentified aliphatic	69+55	MCC	0.16	0.27	0.15	0.15	0.14
23.60	branched alkane	57+71	MCC	0.08	0.08	0.06	0.05	0.08
2.36	alkene C06 (T)	55+69	MCC	1.83	2.18	1.64	1.70	1.51
4.90	alkene C08	(55)69	MCC	0.38	0.22	0.41	0.14	0.14
7.07	alkene C09	55+69	MCC	0.46	0.73	0.58	0.57	0.63
9.28	alkene C10	55+69	MCC	0.58	0.66	0.51	0.54	0.60
11.45	alkene C11	55+69	MCC	0.56	0.66	0.52	0.51	0.46
13.49	alkene C12	55+69	MCC	0.40	0.50	0.34	0.36	0.39
15.41	alkene C13	55+69	MCC	0.29	0.49	0.25	0.31	0.28
17.22	alkene C14	55+69	MCC	0.26	0.49	0.23	0.27	0.25
18.81	alkene C15	55+69	MCC	0.23	0.47	0.20	0.24	0.21
20.45	alkene C16	55+69	MCC	0.21	0.45	0.19	0.22	0.19
21.96	alkene C17	55+69	MCC	0.16	0.43	0.16	0.23	0.18
23.51	alkene C18	55+69	MCC	0.10	0.18	0.09	0.08	0.09
26.20	alkene C20	55+69	MCC	0.06	0.10	0.05	0.05	0.03

29.83	alkene C23	55+69	MCC	0.06	0.10	0.05	0.02	0.03
2.44	alkane C06	57+71	MCC	0.53	0.87	0.75	0.76	0.75
3.43	alkane C07	57+71	MCC	0.65	0.67	0.72	0.79	0.80
5.11	alkane C08	57+71	MCC	0.13	0.15	0.12	0.10	0.11
7.25	alkane C09	57+71	MCC	0.10	0.13	0.10	0.10	0.12
9.48	alkane C10	57+71	MCC	0.13	0.12	0.09	0.08	0.13
11.53	alkane C11	57+71	MCC	0.28	0.15	0.15	0.09	0.16
13.65	alkane C12	57+71	MCC	0.22	0.21	0.16	0.14	0.18
15.56	alkane C13	57+71	MCC	0.17	0.36	0.15	0.22	0.18
17.35	alkane C14	57+71	MCC	0.20	0.45	0.17	0.25	0.19
18.94	alkane C15	57+71	MCC	0.17	0.34	0.07	0.17	0.14
20.53	alkane C16	57+71	MCC	0.18	0.36	0.11	0.17	0.15
22.05	alkane C17	57+71	MCC	0.20	0.38	0.14	0.26	0.20
23.65	alkane C18	57+71	MCC	0.08	0.08	0.06	0.05	0.08
